# Oriented Antibody Covalent Immobilization for Label-Free Impedimetric Detection of C-Reactive Protein *via* Direct and Sandwich Immunoassays

**DOI:** 10.3389/fchem.2021.587142

**Published:** 2021-06-02

**Authors:** Abiola Adesina, Philani Mashazi

**Affiliations:** ^1^ Department of Chemistry, Rhodes University, Makhanda, South Africa; ^2^ Institute for Nanotechnology Innovation, Rhodes University, Makhanda, South Africa

**Keywords:** C-reactive protein, immunosensor, oriented antibody, impedance immunoassay, boronate ester

## Abstract

The detection and monitoring of biological markers as disease indicators in a simple manner is a subject of international interest. In this work, we report two simple and sensitive label-free impedimetric immunoassays for the detection of C-reactive protein (CRP). The gold electrode modified with boronic acid–terminated self-assembled monolayers afforded oriented immobilization of capture glycosylated antibody (antihuman CRP monoclonal antibody, mAb). This antibody-modified surface was able to capture human CRP protein, and the impedance signal showed linear dependence with CRP concentration. We confirmed the immobilization of anti-CRP mAb using surface sensitive X-ray photoelectron spectroscopy (XPS) and electrochemical impedance. The oriented covalent immobilization of mAb was achieved using glycosylated Fc (fragment, crystallizable) region specific to boronic acid. The direct immunoassay exhibited a linear curve for concentration range up to 100 ng ml^−1^. The limit of detection (LoD) of 2.9 ng ml^−1^, limit of quantification (LoQ) of 9.66 ng ml^−1^, and sensitivity of 0.585 kΩ ng^−1^ ml cm^−2^ were obtained. The sandwich immunoassay was carried out by capturing polyclonal anti-CRP antibody (pAb) onto the CRP antigen immunoreaction. The impedance signal after pAb capture also showed linear dependence with CRP antigen concentration and acted as a CRP antigen detection signal amplifier. The detection of the CRP antigen using sandwich pAb immunoassay improved LoD to 1.2 ng ml^−1^, LoQ to 3.97 ng ml^−1^, and enhanced the sensitivity to 0.885 kΩ ng^−1^ ml cm^−2^. The real sample analysis, using newborn calf serum, showed excellent selectivity and % recovery for the human CRP ranging from 91.2 to 96.5%. The method was reproducible to 4.5% for direct immunoassay and 2.3% for sandwich immunoassay.

## Introduction

C- reactive protein (CRP) is a pentraxin family of protein secreted in the liver in response to interleukin 6 (IL-6). The concentration of CRP increases about 1,000 fold in response to injury, inflammation, and tissue damage (Quershi et al., 2009; Qureshi et al., 2012; Boonkaew et al., 2019). CRP is therefore a well-known biomarker for inflammation and tissue damage (Libby et al., 2002). CRP has been identified as the most specific biomarker for inflammation that can independently predict the risk of myocardial infarction (Pepys and Hirschfield, 2003; Hansson, 2005; Casas et al., 2008). The American Heart Association (AHA) and the Center for Disease Control and Prevention (CDC) reported (Verma and Yeh, 2003; Hu et al., 2006; Yang et al., 2009; Chinnadayyala et al., 2019) that individuals with CRP concentrations of less than 1.0 μg ml^−1^ (1.0 ng μl^−1^) are at low risk of cardiovascular disease, while those that have CRP concentrations between 1.0 and 3.0 μg ml^−1^ (1.0 and 3.0 ng μl^−1^) are at moderate risk of cardiovascular disease. Individuals with more than 3.0 μg ml^−1^ (>3.0 ng μl^−1^) CRP concentrations are at high risk of cardiovascular disease. Therefore, monitoring the concentration of CRP in the body and quantifying the amounts may help reduce and predict the risk of cardiovascular disease (CVD) (Sonuç Karaboğa and Sezgintürk, 2018). In the clinical laboratories, enzyme-linked immunosorbent assay (ELISA), turbidimetry, and nephelometry are the major methods used for quantifying the concentration of CRP in human samples (Dominici et al., 2004; Clarke et al., 2005; Parra et al., 2005; Vilian et al., 2019). Although ELISA is a sensitive technique, it is time-consuming and expensive and requires bulky equipment and highly skilled personnel for performing the analysis and result interpretation (Ding et al., 2013). These requirements make ELISA technique not suitable for diagnosis, especially in low-resource settings. An alternative method that offers improved clinical diagnosis of CVD and offers quantitative analysis in a cheap, easy, and fast manner is desirable. The use of electrochemical immunoassays is the promising method that can overcome the ELISA drawbacks, and the detection signal output can be digitized.

Electrochemical immunoassays have received a lot of attention in the field of clinical diagnosis, owing to their higher analytical efficiency as well as the unique advantages of high sensitivity, fast response, portability, low cost, simple instrumentation, and ease of miniaturization (Yu and Wang, 2010; Zhang et al., 2011; Dong et al., 2019; Rong et al., 2019). The electrochemical impedance spectroscopy (EIS) is a powerful technique to investigating a wide variety of electrochemical systems. It is an effective tool for sensing the formation of antibody–antigen affinity reactions occurring on the electrode surface by probing the interfacial properties (Vogt et al., 2016). EIS-based immunosensors are nondestructive and require no labeling of the antibody for signal generation (Repo et al., 2002; Chowdhury et al., 2017). Electrochemical methods are based on the interaction between the antibody and the antigen or aptamer and protein interactions (Wang et al., 2017; Kowalczyk et al., 2018). The results are direct in that the affinity interactions between the antibody and antigen can be directly detected using the changes in charge transfer resistance (R_CT_) (Rodriguez et al., 2005; Bogomolova et al., 2009). The effective immobilization of biomolecules is a major factor in the fabrication of electrochemical impedimetric immunosensors. The sensitivity of an electrochemical impedimetric immunosensor toward the antigen can be enhanced by increasing the loading and the orientation of the capture antibodies. The immobilized capture antibodies must be oriented to allow for the optimum binding of the antigen, that is, with the antigen binding site away from the solid support (Makaraviciute and Ramanaviciene, 2013; Vashist and Luong, 2018). Antibody immobilization *via* adsorption results in various random orientations such as head-on, end-on, and side-on. Head-on antibody orientation results in the Fab (antigen-binding fragment) region attached and blocking the antigen binding. The end-on antibody orientation is *via* the Fc region attachment with antigen binding exposed, and side-on results in the Fc and Fab regions attached leaving at least one Fab antibody arm exposed for antigen binding. A method that seeks to control the orientation and exposes the antigen binding sites is of paramount importance. There are several methods for oriented immobilization of antibodies and these are streptavidin–biotin, C-terminus Fc targeting with amine surfaces, and boronic ester–glycoprotein reaction. Boronic acid and Fc glycoprotein reaction has attracted our research attention due to the high reactivity and can afford maximum antigen binding with both antigen sites exposed.

In this work, we investigate a method of immobilizing monoclonal anti-CRP antibody (mAb) onto the gold electrode surface modified *via* self-assembled monolayer (SAM) of 4-mercaptobenzoic acid (MBA), Au-MBA SAM. 4-aminophenylboronic acid (APBA) was attached *via* amide coupling onto Au-MBA SAM, to yield Au-MBA-APBA SAM. The use of Au-MBA-APBA SAM is to our knowledge for the first time reported for the immobilization of anti-CRP monoclonal antibody (anti-CRP mAb) using boronate ester. The surface analysis using electrochemical cyclic voltammetry, impedance spectroscopy, and X-ray photoelectron spectroscopy was used to confirm the fabrication of gold electrode and the attachment of anti-CRP mAb and blocking nonspecific binding sites with glucose to yield Au-MBA-APBA-mAb/glucose immunosensor. The fabricated impedimetric immunosensor was evaluated for the detection of human CRP antigen in direct and sandwich immunoassays.

## Experiment

### Materials and Reagents

Monoclonal mouse antihuman CRP (MCA5880G, mAb capture antibody), polyclonal goat antihuman CRP (1707-0189G, pAb detection antibody), and native human CRP (1707-2029) were purchased from AbD Serotech. Potassium ferricyanide (K_3_Fe(CN)_6_), potassium ferrocyanide (K_4_Fe(CN)_6_), potassium chloride (KCl), 4-mercaptobenzoic acid (MBA), 4-aminophenylboronic acid (APBA), N-hydroxysuccinimide (NHS), 1-ethyl-3 (3-dimethyl aminopropyl)-carbodiimide (EDC), glucose (blocking reagent), and newborn calf serum (NCS) were purchased from Sigma-Aldrich. Phosphate buffered saline (PBS, 10 mM) solutions of pH 8.0 and 7.4 were prepared using 10 mM KH_2_PO_4_:K_2_HPO_4_ and 0.15 M NaCl; the pH adjustments were conducted using 0.10 M of either HCl or NaOH. All chemicals were of analytical grade. Ultrapure water with a resistivity of 18.2 MΩ cm (at 25°C) was obtained from Milli-Q water purification system and used throughout the experiment.

### Equipment

The electrochemical analysis was carried out using the instrument specifications reported in the Electronic Support Information (ESI). The X-ray photoelectron spectroscopy surface analysis setup reported (Nxele et al., 2015) was followed in this work; the data analysis and fitting of the high resolution spectra interpretation were accomplished using the National Institute of Standards and Technology (NIST) database (Naumkin et al., 2012). The XPS high resolution peak fitting of various components binding energy values were corrected using carbonaceous (C 1s) set at 284.9 eV.

### Fabrication of the Immunosensor

The clean gold surface was modified using self-assembly monolayer (SAM) method by immersing in an absolute ethanol solution containing 4-mercaptobenzoic (MBA, 5.0 mM) for 24 h. The modified gold surface was represented as Au-MBA SAM. The Au-MBA SAM electrode was removed from the thiol solution and rinsed with water and ethanol to remove the physically adsorbed MBA molecules. The terminal -COOH group of the MBA SAM reacted with the amino functional group of 4-aminophenylboronic acid (APBA) using amide coupling reaction. The carboxylic acid of the Au-MBA SAM was activated in 0.40 M of EDC and 0.10 M of NHS in 10 mM PBS pH 7.4 solution for 2 h. The electrode was further rinsed with water and dried with argon gas. The NHS-/EDC-activated electrode was then immersed in pH 7.4 PBS solution containing 25 mM 4-aminophenylboronic solution. After 6 h, the resulting phenylboronic acid electrode, represented as Au-MBA-APBA SAM, was rinsed with water and ethanol to remove unreacted 4-aminophenylboronic acid. The Au-MBA-ABPA SAM was further dried in continuous flow of argon atmosphere. The capture mouse antihuman CRP monoclonal antibody (mAb) was immobilized onto the Au-MBA-APBA SAM to give the Au-MBA-APBA-mAb. The Au-MBA-APBA SAM was immersed into the 10 μl mouse antihuman CRP at pH 7.4 solution (30 μg ml^−1^) at 4°C for 24 h. The antibody-modified gold electrode was washed with PBS (pH 7.4) to remove the unbound antibodies. The unreacted boronic acid sites were blocked by reacting with glucose solution (30 μg ml^−1^ in pH 7.4 PBS) at room temperature for 2 h to give Au-MBA-APBA-mAb/glucose and was stored at 4°C before use.

### Assay Procedure for Detection of Native Human CRP Using Au-MBA-APBA-mAb/Glucose

To assess the analytical performance of the Au-MBA-APBA-mAb/glucose immunosensor, 20 µL solution of CRP antigen with different concentrations ranging from 10 to 400 ng ml^−1^ in PBS (pH 7.4) was applied to the electrode surface. The Au-MBA-APBA-mAb/glucose electrode was incubated for 1 h at room temperature in the various CRP antigen concentrations. Functionalized electrode was reused several times, and the fresh Au-MBA-APBA-mAb/glucose surfaces were obtained by exposing the electrode to 0.1 M HCl solution. The cyclic voltammogram and the impedance were measured and found to be the same as that of a freshly prepared Au-MBA-APBA-mAb/glucose. All measurements were performed in triplicates. The analysis and fitting of the impedance data (Nyquist plot representation) was accomplished using the Randles–Sevcik equivalent circuit. The topology of the circuit contained a solution resistance (R_S_) which was connected in series to a parallel combination of charge transfer resistance (R_CT_) and capacitance (double layer, C_DL_ or constant phase element, CPE) with Warburg impedance (Zw) in the series to the R_CT_. The fitted data was accepted if the %error was less than 5%.

The change in total charge transfer resistance (ΔR_CT_) for each concentration was calculated using Eq. 1:

$$\Delta R_{CT}\;=\;R_{CT\left(Ab-Ag\right)\;}-\;R_{CT\left(Ab\right)}$$ (1)

where R_CT(Ab)_ is the charge transfer resistance of the Au-MBA-APBA-mAb/glucose before the CRP antigen immunoreaction and R_CT(Ab-Ag)_ is the value of the Au-MBA-APBA-mAb/glucose after an immunoreaction with the CRP antigen. Also for sandwich immunoassay, the R_CT(Ab-Ag)_ represented the charge transfer after CRP antigen and polyclonal antibody immunoreaction.

## Results and Discussion

### Fabrication of Anti-CRP Immunosensor

**SCHEME 1:**
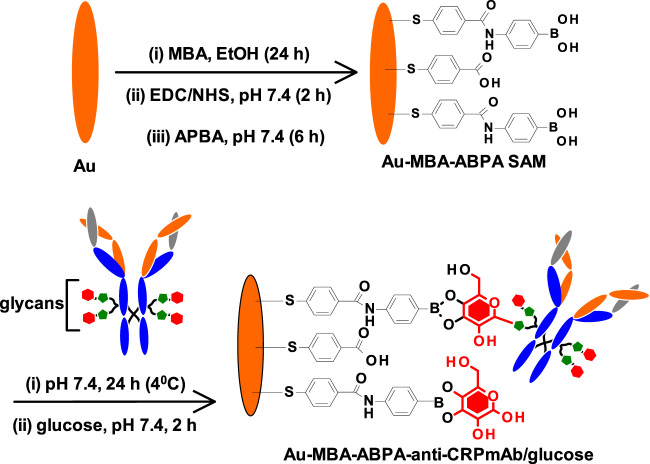
Step-by-step modification of gold surface to form MBA-APBA SAM and the immobilization of monoclonal antihuman CRP antibody.

The fabrication of the CRP-sensing gold surface was modified following the step-by-step procedure shown in Scheme 1 and as described in the experimental section above. The method followed was chosen to allow for oriented immobilization of the monoclonal mouse antihuman CRP antibody (mAb) *via* site-specific glycosylated (glycans) and boronic acid reaction. The unreacted boronic acid reactive surfaces from the Au-MBA-APBA-mAb were blocked with glucose. After glucose reaction, the immunosensor was represented as Au-MBA-ABPA-mAb/glucose. The step-by-step modification of gold surfaces was followed using electrochemistry and X-ray photoelectron spectroscopy (XPS).

### Electrochemical Characterization of the Immunosensor Fabrication

**FIGURE 1:**
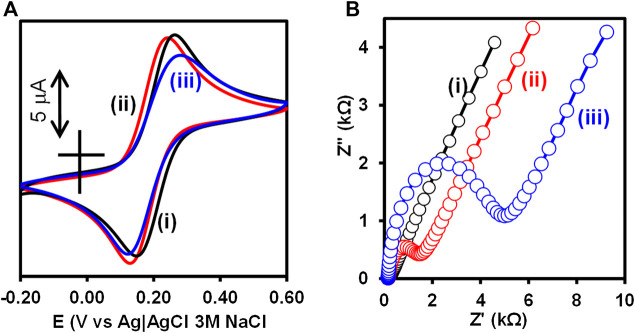
**(A)** Cyclic voltammograms and **(B)** Nyquist plot of (i) bare Au, (ii) Au-MBA-APBA SAM, and (iii) Au-MBA-APBA-mAb/glucose in (1:1) 2 mM K_3_Fe(CN)_6_: K_4_Fe(CN)_6_ solution containing 0.10 M KCl.

Cyclic voltammetry (CV) and electrochemical impedance spectroscopy (EIS) were used to confirm the step-by-step fabrication of the Au-MBA-APBA-mAb/glucose immunosensor. The inhibition of electron transfer properties of the bare gold surface upon modification in the presence of redox probe, [Fe(CN)_6_]^3−/4−^, was use as a measure of surface functionalization. Figure 1 shows (A) CV and (B) EIS of (i) bare Au, (ii) Au-MBA-APBA SAM, and (iii) Au-MBA-APBA-mAb/glucose in (1:1) 2 mM K_3_Fe(CN)_6_:K_4_Fe(CN)_6_ solution containing 0.1 M KCl. The cyclic voltammogram of the bare electrode, in Figure 1Ai, exhibited a reversible redox couple due to [Fe(CN)_6_]^3−/4−^ with peak-to-peak separation (∆E) of 0.115 V. For Au-MBA SAM in Supplementary Figure S1Aii, an increase in ΔE from 0.115 to 0.167 V was observed due to electrostatic repulsion between the negatively charged carboxyl group and negatively charged [Fe(CN)_6_]^−3/−4^. The redox couple was not completely blocked as the Au-MBA SAM was still permeable to solution ions. A decrease in ∆E from 0.167 to 0.113 V was observed when amide coupling between Au-MBA SAM and 4-aminophenylboronic acid occurred, Au-MBA-APBA SAM in Figure 1Aii. The decrease in the peak-to-peak separation at the Au-MBA-APBA SAM was due to the coupling of 4-aminophenylboronic acid and neutralization of the COO^−^ functional group of Au-MBA SAM. The deprotonation of COO^−^ at Au-MBA SAM is due to the high pH conditions (pH 7.4) of the redox probing species solution, [Fe(CN)_6_]^3−/4−^, and the pKa of 5.8 for the carboxylic acid functional group of Au-MBA SAM. Upon amide coupling of the phenylboronic acid, the neutral monolayer resulted due to the fact that the pKa of the boronic acid (OH) functional group is 8.83. At the Au-MBA-APBA-mAb/glucose, in Figure 1Aiii, a slight decrease in current density was observed and due to the blocking behaviour and insulating properties of the mAb and glucose. In addition an increase in ∆E from 0.113 to 0.188 V was observed. The fabrication of Au-MBA-APBA-mAb/glucose resulted in a decrease in peak current density and an increase in ∆E in cyclic voltammogram. This confirms the successful immobilization of the mAb and the blocking of nonspecific binding sites by glucose.

The electrochemical impedance spectroscopy was used to monitor and further confirm the immobilization of monoclonal anti-CRP antibody as this is a more sensitive technique than the cyclic voltammetry. The bare gold electrode exhibited a small semicircle at high frequency region with a charge transfer resistance (R_CT_) value of 60.1 Ω, in Figure 1Bi. An increase in R_CT_ (3.10 kΩ) was obtained after the formation of the MBA SAM on the Au-electrode (Au-MBA SAM), in the ESI Supplementary Figure S1Aii. The increase in R_CT_ was an indication of the electrostatic repulsion between the negatively charged carboxyl (COO^−^) groups of the Au-MBA SAM and the negatively charged [Fe(CN)_6_]^3−/4−^ ions. This reduces the ability of the [Fe(CN)_6_]^3−/4−^ ions to reach the underlying gold electrode surface and has been reported before (Moreno-Guzmán et al., 2012). Upon further modification with aminophenylboronic acid (Au-MBA-APBA SAM), in Figure 1Bii, a drastic decrease in R_CT_ from 3.10 to 1.10 kΩ was observed. This could be attributed to the neutralization of the negative charge carboxylic group upon reaction with the amino group of 4-aminophenylboronic acid. Upon the immobilization of mAb and blocking the nonspecific binding site with glucose, Au-MBA-APBA-mAb/glucose modified electrode, in Figure 1Biii, the R_CT_ increased from 1.10 to 4.09 kΩ due to the insulating properties of the immobilized monoclonal antibody and the glucose. This results in slow rate of electron transfer properties of the gold electrode and increase in the charge transfer resistance. The optimum concentration of glucose (30 μg ml^−1^) for the blocking was used and did not have an effect on the capture of the biomarker (human CRP protein). Table S1 shows the summary of the CV and EIS parameters. The EIS and CV results confirm the successful modification of the electrode surface. Additional characterization of the gold electrode surface modification was carried out using surface sensitive and quantitative capabilities of the X-ray photoelectron spectroscopy (XPS).

### XPS Characterization of the Immunosensor Fabrication

**FIGURE 2:**
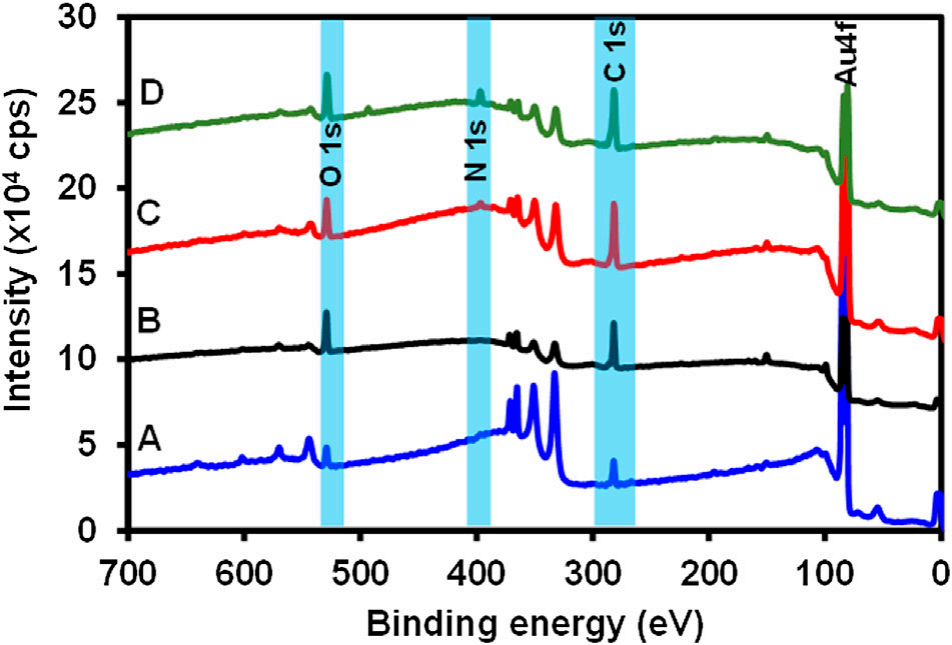
The survey spectra of **(A)** bare Au, **(B)** Au-MBA SAM, **(C)** Au-MBA-APBA SAM, and **(D)** Au-MBA-APBA-mAb/glucose.

XPS is a highly sensitive, versatile, and quantitative surface characterization technique that gives insight into the type of bonding and interactions that exist on a surface (Korin et al., 2017). The elemental and the atomic composition can be evaluated using the XPS survey spectra, thus giving this technique the quantitative analytical capability (Etorki et al., 2012). To ascertain the successful fabrication of the immunosensor, different modification stages of the immunosensor design were characterized using XPS. Figure 2 shows the survey spectrum of (**A**) bare Au, (**B**) 4-mercaptobenzoic acid modified surface (Au-MBA SAM), (**C**) 4-aminophenylboronic acid modified (Au-MBA-APBA SAM), and (**D**) anti-CRP antibody modified electrode (Au-MBA-APBA-mAb/glucose). The survey spectra of the bare Au revealed the presence of major peaks due to gold (Au 4f, Au 4p, and Au 4d) and other elements such as chromium (Cr), silver (Ag), oxygen (O1s), and carbon (C1s) peaks. The presence of carbon and oxygen peaks could be due to the washing of the bare Au surface using ethanol and air before measurement. The presence of silver and chromium peak is attributed to the surface coating of the gold-coated quartz crystal to obtain a smooth gold thin layer with the Cr underlayer and conducting silver. The atomic percentage (at %) of carbon and oxygen were found to be 45.2 and 12.2%, respectively, for the bare electrode (Figure 2A). Upon formation of the self-assembled monolayer of MBA (Figure 2B), a significant increase in both for carbon (from 45.2 to 60.4%) and oxygen (from 12.2 to 18.9%) was observed. The observed increase in both carbon and oxygen for the MBA SAM accounts for the successful formation of the self-assembled monolayer. Furthermore, the silver composition percent decreased from 5.67 to 1.67%. This is attributed to addition of the SAM layer on the electrode surface thereby causing a reduction of the silver concentration. The covalent immobilization of 4-aminophenylboronic acid onto Au-MBA SAM (Figure 2C, Au-MBA-ABPA SAM) resulted in the appearance of nitrogen (N 1s). The N 1s peak is attributed to amide (-CONH-) moiety that formed during the amide coupling of the 4-aminophenylboronic acid to the EDC-/NHS-activated COOH-terminated surface of the Au-MBA SAM. The presence of boron (B 1s) element was also seen at the APBA-modified electrode which is an indication of the successful modification of the electrode with APBA. A significant increase in the intensity of the N 1s peak was observed upon boronate ester formation of the Au-MBA-ABPA SAM with the Fc saccharides of the anti-CRP monoclonal antibody to yield an Au-MBA-ABPA-mAb surface (Figure 2D). The boronate ester reactions allow for the oriented immobilization of the antibody exposing the antigen-binding site. The increase in N 1s intensity at Au-MBA-ABPA-mAb is attributed to the immobilization of the antibody containing numerous peptide bonds.

**FIGURE 3:**
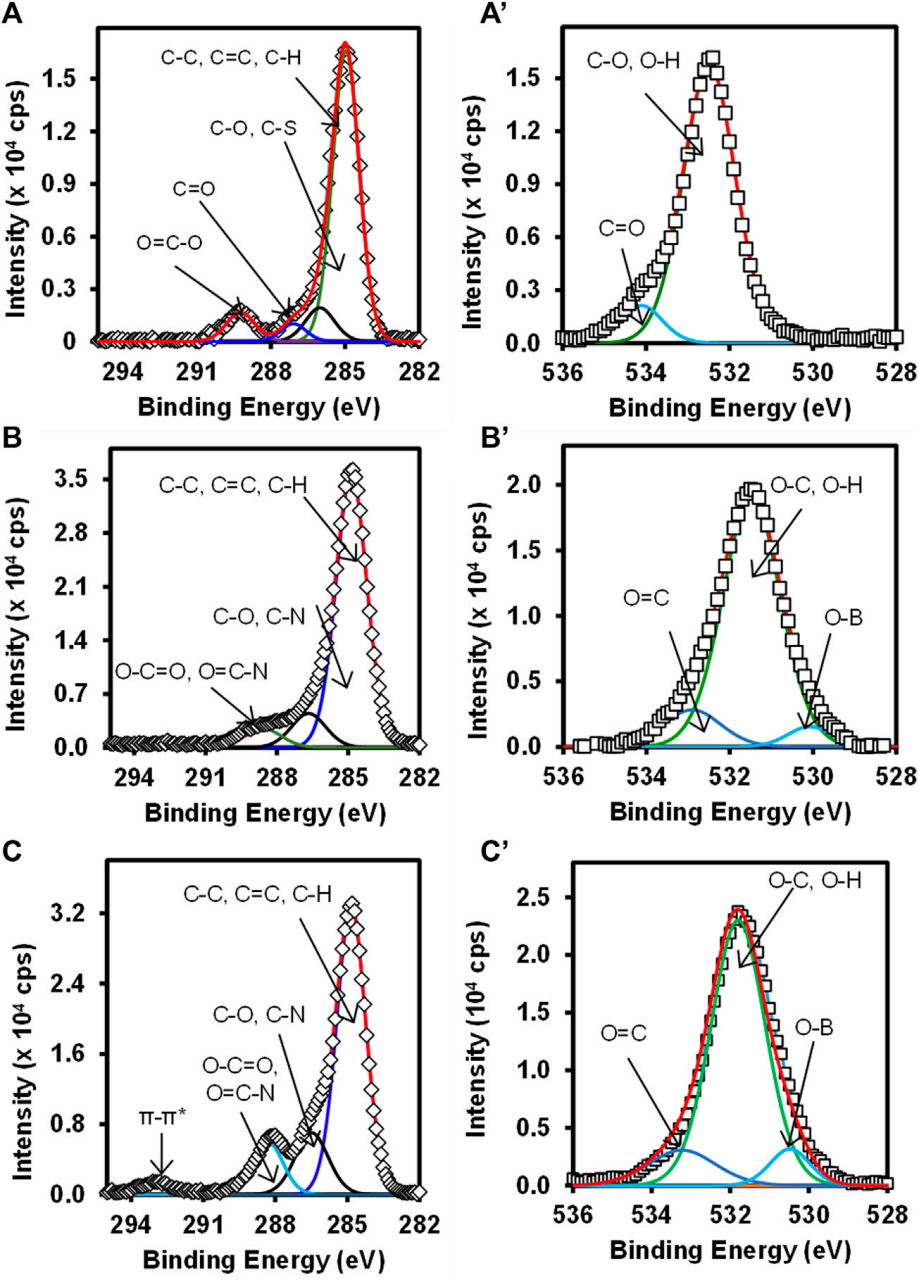
High-resolution spectra of C 1s **(A)** Au-MBA SAM, **(B)** Au-MBA-APBA SAM, and **(C)** Au-MBA-APBA-mAb/glucose and O 1s **(A’)** Au-MBA SAM, **(B’)** Au-MBA-APBA SAM, and **(C’)** Au-MBA-APBA-mAb/glucose.

Figure 3 shows the high-resolution C 1s (**A**) Au-MBA SAM, (**B**) Au-MBA-APBA SAM, and (**C**) Au-MBA-APBA-mAb/glucose and O 1s (**A’**) Au-MBA SAM, (**B’**) Au-MBA-APBA SAM, and (**C’**) Au-MBA-APBA-mAb/glucose. The deconvoluted C 1s high-resolution spectra gave the chemical environment of carbon present on the surface and according to the material used for the modification. For Au-MBA-SAM (Figure 3A), four components were observed at 284.9, 286.0, 287.1, and 289.1 eV. The components observed at 284.9 eV was assigned to C-C, C=C, and C-H (Gruian et al., 2012; Maya Girón et al., 2016). The component at 286.0 eV was assigned to C-O and C-S from Au-MBA SAM. The component at 287.1 eV was assigned C=O. The component at 289.1 eV is due to O-C=O (Barriet et al., 2007; Fabre and Hauquier, 2017; Liu et al., 2016). The C 1s high resolution of the Au-MBA-APBA SAM surface (Figure 3B) shows three components at 284.9, 287.5, and 288.5 eV, and their assignments are similar to the Au-MBA SAM except the component at 288.5 eV is assigned N-C=O (amide bond) (Song and Yoon, 2009). The C 1s spectrum of Au-MBA-APBA-mAb/glucose surface was deconvoluted and four components were synthesized (Figure 3C) at 284.9, 287.0, 288.6, and 293.2 eV. The component at 284.9 eV is assigned as the above. The component at 287.0 eV corresponds to C-O, C-S, and C-N carbons of the protein backbone (Iucci et al., 2004). The third component at 288.6 eV is assigned to the carboxyl (O-C=O) and peptide carbon (N-C=O) (Lebugle et al., 1996) due to various amide bonds on the backbone of the antibody. The last component seen at 293.2 eV is the shake-up which is associated with carbon in the aromatic ring (Dave et al., 2015). The high-resolution O 1s for (**A’**) Au-MBA-SAM, (**B’**) Au-MBA-APBA SAM, and (**C’**) Au-MBA-APBA-mAb/glucose surfaces is also presented in Figure 3.

The deconvolution of the O 1s Au-MBA-SAM in Figure 3A’ was fitted into two components. The component at 532.5 eV is assigned to the O-C and O-H of the mercaptobenzoic acid (Au-MBA SAM). The second component at 534.4 eV is due to the carbonyl oxygen (C=O) of the carboxyl group (Caprioli et al., 2013). The deconvolution of the O 1s spectrum in Figures 3B’,C’ for Au-MBA-APBA SAM and Au-MBA-APBA-mAb/glucose both fitted into three components. The components were observed at binding energies at 530.2, 531.6, and 532.7 eV for Au-MBA-APBA SAM corresponding to O-B, (O-C, O-H), and O=C, respectively. For the Au-MBA-ABPA-mAb/glucose, the binding energies were at 530.6, 531.6, and 533.1 eV corresponding to O-B, (O-C, O-H) and O=C, respectively, from the MBA-ABPA-mAb/glucose. The binding energy at 531.6 eV can be assigned to O-C and O-H (Whelan et al., 2004). The component centered at approximately 530.2 eV or 530.6 eV is assigned to B-O bond due to boron atom (Kim et al., 2017), and components at approximately 532.7 and 533.1 eV are attributed to the carbonyl oxygen (C=O) present in COOH and amide (HNCO) functional group (Kang et al., 2017). The presence of the observed carbon and oxygen species with their chemical environments confirmed the immobilization of the MBA, MBA-ABPA, and MBA-ABPA-mAb/glucose. There was also an increase in the intensity after each immobilization signifying the attachment of the various materials.

**FIGURE 4:**
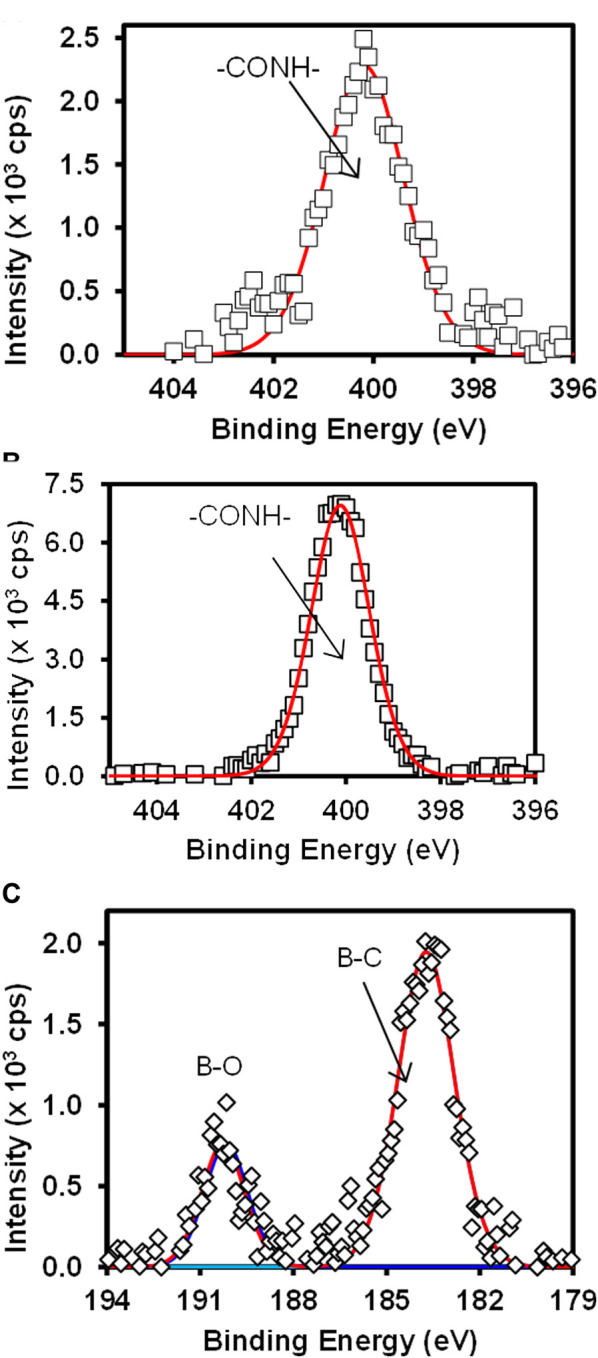
High-resolution spectra of N 1s for **(A)** Au-MBA-APBA SAM and **(B)** Au-MBA-APBA-mAb/glucose and **(C)** the high-resolution spectrum of B 1s of Au-MBA-APBA SAM.

Figure 4 shows the high-resolution N 1s for (**A**) Au-MBA-APBA SAM and (**B**) Au-MBA-APBA-mAb/glucose surfaces and (**C**) the boron (B 1s) for Au-MBA-ABPA SAM. The high resolution of N 1s for Au-MBA-APBA SAM and Au-MBA-APBA-mAb/glucose in Figures 4A,B shows single component at 400.2 and 400.3 eV, respectively, attributed to the amide (CONH) bond (Costello et al., 2012; Eissa and Zourob, 2017). It was interesting to observe a remarkable increase in N 1s intensity from 2.5 × 10^3^ cps to 7.5 × 10^3^ cps after the immobilization of mAb/glucose on the Au-MBA-APBA SAM. This confirmed that blocking by glucose did not result in the displacement of mAb. Two major and distinct components were observed from the high-resolution spectrum of B 1s in Figure 4C and were centered at 183.9 and 190.5 eV. These components are indicative of the presence of boron element with two chemical environments. The component at 183.9 eV was assigned to B-C bond, while the other component at 190.5 eV was assigned to B-O bond (Yang et al., 2016). The presence of boron and nitrogen peaks on the Au-MBA-APBA SAM confirms the successful amide coupling of APBA onto an Au-MBA SAM. However, the disappearance of boron element was observed with the Au-MBA-APBA-mAb/glucose electrode and due to the bulky mAb and glucose boronate ester formation. This observation could further be attributed to the macro size of the mAb which confirms the successful immobilization of the antibody onto the MBA-APBA SAM functionalized gold surface. The quantitative analysis results are summarized in Supplementary Table S2 (ESI).

### Detection of Human CRP Antigen

**FIGURE 5:**
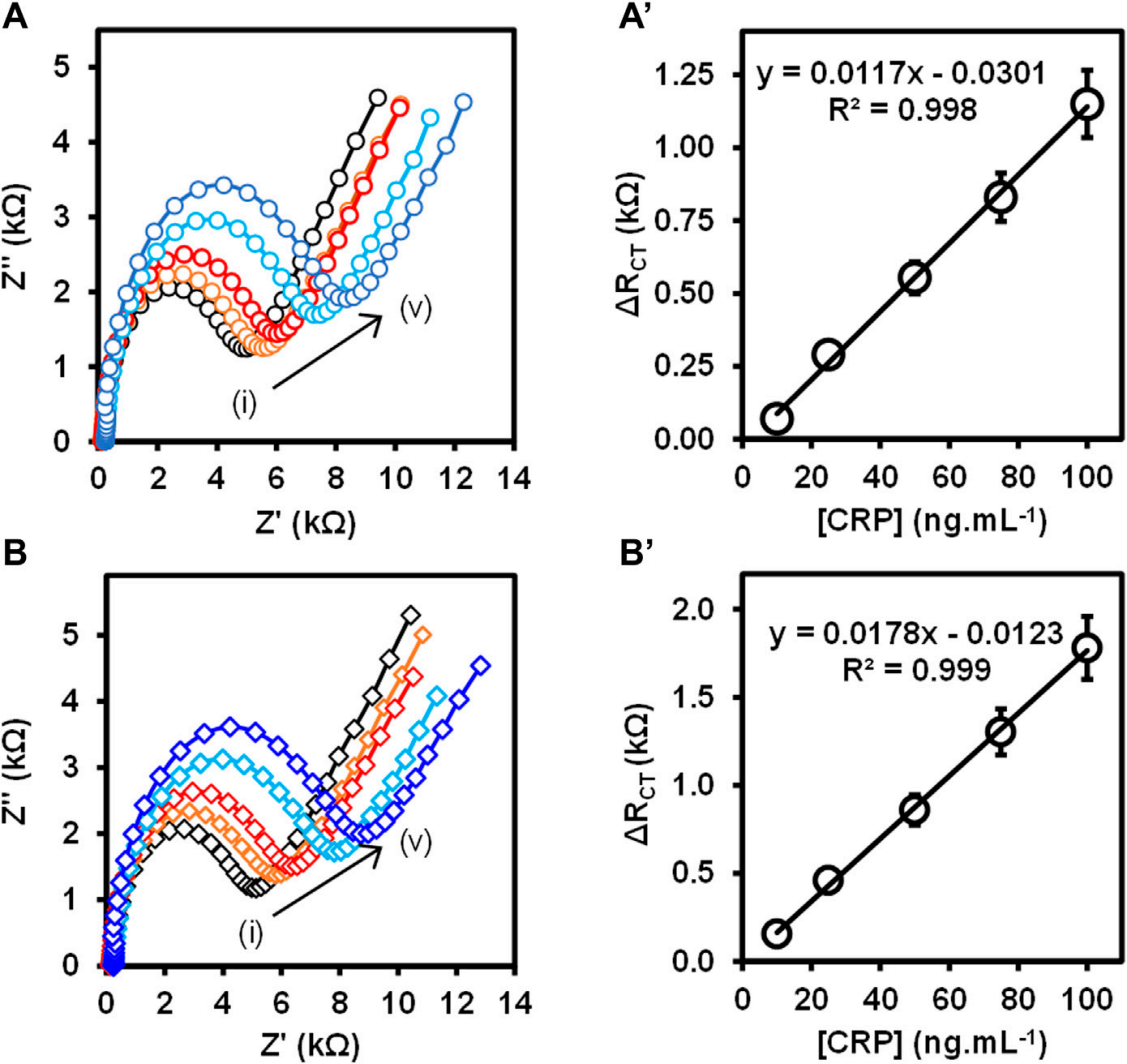
**(A,B)** Nyquist plots and **(A’,B’)** the corresponding calibration curves at varied CRP antigen concentrations (i) 10 ng ml^−1^, (ii) 25 ng ml^−1^, (iii) 50 ng ml^−1^, (iv) 75 ng ml^−1^, and (v) 100 ng ml^−1^ for the **(A)** direct and **(B)** sandwich immunoassays. (n = 3, SD ≤ 9.0%).

The Au-MBA-APBA-mAb/glucose immunosensor was used to detect CRP antigen at different concentrations ranging from 10 to 400 ng ml^−1^ using direct and sandwich immunoassay formats. The total ΔR_CT_ values obtained with their relative standard deviation (% RSD) for the direct and sandwich assays at different CRP antigen concentrations are shown in Table S3 (ESI). Figure 5 shows the Nyquist plots (**A**) and (**B**) with their corresponding linear graphs of ΔR_CT_ against CRP antigen concentrations (**A’**) and (**B’**) for the (**A**) direct and (**B**) sandwich immunoassays. For the direct immunoassay, the anti-CRP mAb/glucose immunosensor was exposed to the CRP antigen, and the impedance spectroscopy was measured. The increase in the charge transfer resistance (R_CT_) was observed with increasing CRP antigen concentrations. The change in total charge transfer resistance (ΔR_CT_) for each CRP antigen concentration was calculated using Eq. 1. ΔR_CT_ increased linearly with increasing antigen concentration from 10 to 100 ng ml^−1^ and the linear regression equation was ΔR_CT_ = 0.0117 [CRP antigen] −0.0301 and *R* ^2^ = 0.998 (*n* = 3), shown in Figure 5A’. However, at higher antigen concentration, a decrease in the R_CT_ value was observed between 200 and 400 ng ml^−1^ in Supplementary Figure S2, ESI. This is attributed to excess amount of CRP antigen competing for the same binding site, thus leading to partial binding. Upon rinsing the partially bound CRP antigen washes off. This phenomenon has been observed before (Songjaroen et al., 2016) for immunosensors based on antigen–antibody affinity detection. The high concentration of CRP antigen results in the reduction of the binding efficiency between the immobilized anti-CRP monoclonal antibody and CRP antigen. The sandwich assay using polyclonal anti-CRP antibody (pAb) that recognizes different epitopes on the captured CRP antigen was used for the signal amplification. The sandwich immunoassay involved two steps: 1) the capture of CRP antigen followed by rinsing and 2) exposing the captured CRP antigen to pAb. After rinsing, the impedance spectroscopy was recorded and R_CT_ was obtained after fitting the Nyquist plot using the Randles–Sevcik equivalent circuit.

Figure 5B shows the Nyquist plots for the detection of different concentrations of CRP antigen (10–100 ng ml^−1^) for the sandwich immunoassay. The pAb was kept constant at 5.0 μg ml^−1^. The increase in the semicircle (R_CT_) was observed with increasing concentration of CRP antigen confirming that the binding occurred between the surface bound mAb antibody and CRP antigen and lastly the pAb, resulting in the mAb/CRP/pAb immunoreaction. The calibration curve is shown in Figure 5B’ for the change in total charge transfer resistance according to Eq. 1, given above. The linear regression equation was ΔR_CT_ = 0.0178 [CRP antigen] −0.0123 with *R* ^2^ = 0.999 (*n* = 3) was obtained for the sandwich immunoassay. The sensitivity of the direct immunoassay was 0.585 kΩ ng^−1^ ml cm^−2^ and increased for the sandwich immunoassay to 0.885 kΩ ng^−1^ ml cm^−2^. The increase in the sensitivity for the sandwich assay was attributed to the enhancement in the signal generated by the pAb as observed in Figure 5B’. The key analytical parameters for evaluating the developed method and the performance are the limit of detection (LoD) and limit of quantification (LoQ). The LoD and the LoQ were calculated using the IUPAC method of 3 × SD/m and 10 × SD/m, respectively, where m is the slope of the calibration curve and SD is the standard deviation of the blank measurements without the presence of the CRP antigen. The LoD and LoQ for the direct immunoassay were calculated to be 2.90 and 9.66 ng ml^−1^, respectively, and for the sandwich immunoassay, the LoD was 1.20 and LoQ was 3.97 ng ml^−1^. The lower LoD and LoQ for the sandwich immunoassay were attributed to the high sensitivity and the signal amplification. The immunosensor is highly sensitive and shows lower LoDs and LoQs than the method used for the detection of CRP antigen and summarized in Table 1. The developed immunoassay for the detection of CRP is comparable in terms of the LoDs and linear concentration range to the various methods reported in Table 1. The enhanced detection and better analytical properties of the designed immunosensor is further attributed to the oriented immobilization of the capture monoclonal anti-CRP antibody (mAb). This method of immobilizing mAb *via* Fc-specific region using boronate ester allows for the CRP antigen site to be exposed to the analyte (CRP antigen). Lower LoDs 1.20 ng ml^−1^ and 2.90 ng ml^−1^ and LoQs 3.97 ng ml^−1^ and 9.66 ng ml^−1^ for sandwich and direct immunoassays are, respectively, reported for the detection of human CRP antigen. The LoDs were lower than those reported in literature (Rajesh et al., 2010; Gupta et al., 2014) and higher than the values reported by Lv et al. (2017). The better LoDs reported by Lv et al. (2017) used the quantum dots for fluorescence detection. However, the immunosensor yielded a very narrow range for the detection of human CRP, that is, 10–100 ng ml^−1^. This range falls outside the clinically relevant range for the human CRP which is 1.0–3.0 ng μl^−1^. The previously reported methods have a wider concentration range. The other parameter determined in this work was the sensitivity which was 0.585 kΩ ng^−1^ ml cm^−2^ for the direct immunoassay and 0.885 kΩ ng^−1^ ml cm^−2^ for the sandwich immunoassay. The reason for the observed narrow concentration range is that the capture mAb immobilized onto gold electrode surface reached saturation at human CRP concentrations of 100 ng ml^−1^ for both the direct and sandwich immunoassays. The study, however, paves a way of fabricating stable antibody thin films and their use in the detection of biologically relevant biomarkers.

**TABLE 1:** Analytical parameters for the electrochemical immunosensor for CRP antigen detection compared with reported methods.

Detection method	Assay linear range (ng ml^−1^)	Sens. (kΩ.ng^−1^. mL.cm^−2)^	*R* ^2^	LoD (ng ml^−1^)	LoQ (ng ml^−1^)	Ref
Impedance spectroscopy	10–100^a^	0.585^a^	0.998^a^	2.90^a^	9.66^a^	This work
	10–100^b^	0.885^b^	0.999^b^	1.20^b^	3.97^b^	
Electrochemistry	50–5,000	—	—	11.0	—	Gupta et al. (2014)
Electrochemistry	8.5–9,120	—	—	3.50	—	Rajesh et al. (2010)
Electrochemistry	1.56–400	—	—	0.46	—	Lv et al. (2017)
Electrochemistry	0–100	—	—	2.2	—	Fakanya and Tothill. (2014)
Electrochemistry	0.5–200	—	—	1.2	—	Zhou et al. (2010)
Electrochemistry	0.2–80	—	—	0.040	—	Zhang et al. (2016)

a is the measurement using the direct immunoassay.

b is the measurement using sandwich immunoassays.

### Specificity and Reproducibility of the Fabricated Immunosensor

To assess the specificity of the immunosensor, a control experiment was carried out with the direct interaction of the anti-CRP mAb–modified gold electrode with the polyclonal anti-CRP antibody (pAb). R_CT_ for the anti-CRP mAb was found to be 5.46 kΩ, and after exposure to pAb, the R_CT_ value of 5.48 kΩ showed no change in R_CT_. This confirmed that an increase in the total change in charge transfer resistance (ΔR_CT_) between anti-CRP antibody (mAb) and the CRP antigen in Figure 5 is due to the formation of an immunocomplex. Without the CRP antigen, there was no change in R_CT_ values 5.46 and 5.48 kΩ. The reproducibility of the immunosensor was investigated since it is a crucial factor to be considered in real-life applications. For reproducibility studies, three different immunosensors were prepared independently at the same and varied experimental conditions. The immunosensors were evaluated against the same CRP antigen concentration. The relative standard deviation (% RSD) for the immunosensor was found to be 4.47% for the direct immunoassay and 2.31% for the sandwich immunoassay tested for 50 ng ml^−1^ CRP antigen concentration. This confirmed that the method of designing the CRP antigen sensing was reproducible with about 2.31 and 4.47% variation.

### Real Sample Analysis

The accuracy of the proposed immunosensor toward the detection of CRP antigen was investigated in serum sample through a recovery experiment. The recovery test was carried out in 10% newborn calf serum in PBS (pH 7.4). Two different CRP antigen concentrations (25 and 50 ng ml^−1^) were used for the recovery experiment. The percentage obtained from the recovery test was between 91.18 and 96.47%. The amount recovered was very close to 100%. This shows that the immunosensor can be applied in clinical applications for the analysis of CRP antigen. The use of the newborn calf serum was to mimic the real samples. Human serum would have been ideal and is planned for future investigation. Table 2 shows the summarized information for both the direct and sandwich immunoassays.

**TABLE 2:** Spike and recovery results obtained from the fabricated CRP immunosensor in serum samples.

Sample number	Spiked (ng ml^−1^)	Found (ng ml^−1^)	Recovery (%)
1	25 (direct)	27.30	95.87
2	25 (sandwich)	26.50	96.47
3	50 (direct)	49.90	94.94
4	50 (sandwich)	49.00	91.18

## Conclusion

This work demonstrated the fabrication of electrochemical impedimetric immunosensor for the detection of C-reactive protein antigen. The electrochemical and X-ray photoelectron spectroscopy characterization methods were used to confirm the formation of the self-assembled monolayer of 4-mercaptobenzoic acid, coupling of 4-aminophenylboronic acid *via* amide bond and immobilization of anti-human CRP monoclonal antibody onto gold electrode surface. The increase in intensity of C 1s, O 1s, and N 1s confirmed the immobilization and coupling (*via* amide and boronate ester) of various materials studied. An excellent sensitivity of the immunosensor was obtained to be 0.585 kΩ ng^−1^ ml cm^−2^ for the direct immunoassay. An even better sensitivity was obtained for the sandwich immunoassay to be 0.885 kΩ ng^−1^ ml cm^−2^ and almost double that of the direct immunoassay. The excellent sensitivity was ascribed to the oriented immobilization *via* boronate ester targeting an Fc region of the capture anti-CRP monoclonal antibody. The analytical parameters for the immunosensors for the direct immunoassay were calculated to be 2.90 ng ml^−1^ for LoD and 9.66 ng ml^−1^ for LoQ. For the sandwich immunoassay, the enhanced analytical parameters were 1.20 ng ml^−1^ for the LoD and 3.97 ng ml^−1^ for the LoQ. The immunosensor showed good reproducibility with % RSD of 4.47% for the direct immunoassay and 2.31% for the sandwich immunoassay. The immunosensor shows promising results toward the label-free detection of C-reactive protein. The newborn calf serum analysis showed good % recovery of the actual amounts injected in the sample. The % recovery ranged from 91.18 to 96.47%. Human plasma or blood samples are subject of our continued research for the detection of human CRP antigen as an important biomarker.

## Data Availability

The original contributions presented in the study are included in the article/Supplementary Material; further inquiries can be directed to the corresponding author.
