# Supplementary material for: Oriented Antibody Covalent Immobilization for Label-Free Impedimetric Detection of C-Reactive Protein via Direct and Sandwich Immunoassays
Source: Front Chem. 2021 Jun 2;9:587142. doi: 10.3389/fchem.2021.587142 (PMC8207519; doi:10.3389/fchem.2021.587142)
Supplement: Supplementary file 1 [file DataSheet1.docx]

**Electronic Supplementary Information (ESI)**

**Oriented antibody covalent immobilization for label-free impedimetric detection of C-reactive protein via direct and sandwich immunoassays**

Abiola Adesina,^a^ Philani Mashazi^a,b,^*

^a^Department of Chemistry, ^b^Institute for Nanotechnology Innovation, P. O. Box 94, Rhodes University, Makhanda, 6140, South Africa

*Corresponding Author: Email: [p.mashazi@ru.ac.za](mailto:p.mashazi@ru.ac.za)

**Experimental**

All electrochemical and impedance spectroscopy experiments were carried out using the AUTOLAB PGSTAT 302N Electrochemical Workstation interfaced to a proline desktop computer equipped with a 1.10 version NOVA. A three-electrode system using a gold disk electrode (r = 0.8 mm) purchased from BASi (USA) as working electrodes. Platinum wire as counter electrode and silver/silver chloride (Ag/AgCl) 3M NaCl solution as reference electrode. All cyclic voltammetry (CV) and electrochemical impedance spectroscopy (EIS) experiments were recorded in 2.0 mM [Fe(CN)_6_]^3-/4-^ redox couple in PBS buffer (pH 8.0). CVs were recorded between -0.2 to 0.6 V (Ag/AgCl) 3M NaCl. EIS data were recorded between 10 kHz to 100 MHz at a bias potential of 0.2 V with an amplitude 5 mV rms sinusoidal modulation.

The surface of the gold disk electrode (diameter 1.6 mm, geometric surface area = 0.0201 cm^2^) was polished with different sizes of alumina slurries (1, 0.3 and 0.05 µm) and sonicated in ethanol and distilled water solution for 5 minutes. It was then immersed into the freshly prepared piranha solution (concentrated H_2_SO_4_:H_2_O_2_, v/v 3:1) for 3 min. The polished gold electrode was electrochemically scanned in 0.50 M H_2_SO_4_ aqueous solution within the potential range (-0.20 - 1.5 V) at a scan rate of 0.10 Vs^-1^ until a reproducible reduction peak was obtained. The clean gold electrode was then rinsed with ethanol and dried in an argon atmosphere.

**Results and discussion**

**Figure S1**: (a) CV and (a’) EIS of (i) Au, (ii) Au-MBA SAM and (iii) Au-MBA-APBA SAM and (b) CV and (b’) EIS of (i) Au-MBA-APBA-mAb and (ii) Au-MBA-APBA-mAb/glucose in (1:1) 2 mM K_3_Fe(CN)_6_:K_4_Fe(CN)_6_ solution containing 0.1 M KCl.

**Figure S2:** Nyquist plot showing (a) the decrease in R_CT_ at higher CPR-antigen concentrations (i – ii) 100-400 ng.mL^-1^ for the direct immunoassay, (b) a slight increase in R_CT_ signal after (i) – (ii) for 100 – 400 ng.mL^-1^ for CRP-antigen observed for sandwich immunoassay.

**Figure S3** shows a plot of the change in total R_CT_ (ΔR_CT_) versus the concentration of CRP-antigen for (i) direct immunoassay and (ii) sandwich immunoassay. A linear plot was observed for the CRP-antigen concentrations up to 100 ng.mL^-1^ for both direct and sandwich immunoassays. After 100 ng.mL^-1^, a deviation from linearity was observed and has been discussed in the main manuscript.

**Figure S3:** Calibration curves (ΔRCT vs [CRP]) showing concentrations from 10 - 400 ng.mL^-1^ for (i) direct immunoassay and (ii) sandwich immunoassay.

**Table S1**: Summary of the CV and EIS parameters Au, Au-MBA SAM, Au-MBA-APBA SAM Au-MBA-APBA-mAb and Au-MBA-APBA-mAb/glucose measured in (1:1) 2 mM K_3_Fe(CN)_6_:K_4_Fe(CN)_6_ solution containing 0.1 M KCl.

| Electrode | ΔE (V) | R_CT_ (kΩ) |
| --- | --- | --- |
| **Au** | 0.115 | 0.0601 |
| **Au-MBA SAM** | 0.167 | 3.10 |
| **Au-MBA-APBA SAM** | 0.113 | 1.10 |
| **Au-MBA-APBA-mAb** | 0.158 | 3.38 |
| **Au-MBA-APBA-mAb/glucose** | 0.188 | 4.09 |

**Table S2:** Summary of the component positions, component percentage and their assignments for different stages of the fabricated immunosensor.

| Surfaces | Atoms | Comp. pos. (eV) | Comp.  Assignments | Comp. percent (%) |
| --- | --- | --- | --- | --- |
| Au-MBA SAM | C 1s | 284.6, 285.7  286.7  288.9 | C-C, C=C, C-H  C-O, C-S  O-C=O | 76.39, 10.20  4.75  8.66 |
|  | O 1s | 531.2  532.8 | C-O, O-H  C=O | 87.83  12.17 |
| Au-MBA-APBA | C 1s | 284.6  286.7  288.3 | C-C, C=C, C-H  C-O, C-S, C-N  O-C=O, N-C=O | 81.66  11.51  6.83 |
|  | O 1s | 530.6  532.0  533.4 | B-O  C=O  O-C=O, N-C=O | 82.90  12.20  4.90 |
|  | N 1s | 400.2 | N-C=O | 100 |
|  | B 1s | 184.4  190.7 | B-C  B-O | 76.80  23.20 |
| Au-MBA-APBA mAb/glucose | C 1s | 284.6  286.3  288.0  292.4 | C-C, C=C, C-H  C-O, C-S, C-N  N-C=O, O-C=O  π-π* | 67.93  16.93  12.54  2.60 |
|  | O 1s | 530.5  531.8  533.1 | B-O  C=O  O-C=O, N-C=O | 81.89  10.60  7.51 |
|  | N 1s | 400.3 | N-C=O | 100 |

**Keys:** Comp. (components), pos. (positions, eV)

**Table S3:** Total ΔR_CT_ and relative standard deviation (% RSD, n = 3) obtained from EIS for direct and sandwich immunoassay at different antigen concentrations.

| [CRP-Antigen] (ng.mL^-1^) | Direct immunoassay | | Sandwich assay | |
| --- | --- | --- | --- | --- |
|  | **(ΔR_CT_, kΩ)** | **% RSD** | **(ΔR_CT_, kΩ)** | **% RSD** |
| 10 | 0.070 | 9.42 | 0.156 | 6.07 |
| 25 | 0.290 | 7.71 | 0.460 | 3.14 |
| 50 | 0.554 | 5.84 | 0.860 | 4.23 |
| 75 | 0.833 | 1.68 | 1.304 | 0.86 |
| 100 | 1.154 | 4.83 | 1.782 | 7.21 |
| 200 | 0.920 | 0.84 | 2.020 | 4.34 |
| 400 | 0.410 | 2.43 | 2.670 | 0.65 |

**Procedure for the** **control experiment**

The specificity of the immunosensor was carried out by incubating the (Au-MBA-APBA-mAb/glucose electrode with the polyclonal antibody (5 µg.mL^-1^) for 2 hours. The difference in charge transfer resistance between the mAb/glucose electrode and after the interaction with polyclonal antibody (mAb/glucose/pAb) were recorded. There was no significant change in charge transfer resistance after the incubation with the polyclonal antibody. This indicates that the polyclonal CRP antibody does not reacts directly with the Au-MBA-APBA-mAb/glucose electrode.
